# E3 Ubiquitin Ligase CHIP Inhibits the Interaction between Hsp90*β* and MAST1 to Repress Radiation Resistance in Non-Small-Cell Lung Cancer Stem Cells

**DOI:** 10.1155/2022/2760899

**Published:** 2022-09-20

**Authors:** Bo Tan, Jingwei Zhang, Wen Wang, Haibo Ma, Yuanyuan Yang

**Affiliations:** ^1^Department of Radiotherapy, Affiliated Cancer Hospital of Zhengzhou University & Henan Cancer Hospital, Zhengzhou 450008, China; ^2^Department of Thoracic Surgery, Affiliated Cancer Hospital of Zhengzhou University & Henan Cancer Hospital, Zhengzhou 450008, China

## Abstract

The radiation resistance of cancer stem cells poses a critical obstacle for management of non-small-cell lung cancer (NSCLC). It is interesting to note that E3 ubiquitin ligase CHIP is involved in radiation resistance and stemness phenotypes in NSCLC, while the downstream mechanisms remain elusive. Therefore, this study is aimed at exploring the possible molecular mechanism of E3 ubiquitin ligase CHIP in radiation resistance of NSCLC stem cells. Cancer and adjacent normal tissues of NSCLC patients were collected to determine expression of CHIP, Hsp90*β*, and MAST1. CD133^+^ cells were isolated from the NSCLC tissues and the lung cancer cell line A549 by flow cytometric sorting. Accordingly, downregulated CHIP and upregulated Hsp90*β* and MAST1 were observed in cancer tissues from NSCLC patients and in NSCLC stem cells. Sphere formation assay, colony formation assay, and flow cytometry were performed to examine self-renewal ability, survival, and apoptosis of NSCLC stem cells. An animal model of tumor xenograft was developed in nude mice to observe the tumorigenic ability and radiation resistance of NSCLC stem cells. CHIP overexpression was demonstrated to inhibit the NSCLC stem cell properties and radiation resistance *in vitro* and *in vivo*. Mechanistically, CHIP promoted MAST1 ubiquitination by blocking Hsp90*β* interaction with MAST1, thus inhibiting MAST1 protein stability. Furthermore, CHIP-mediated downregulation of MAST1 protein stability inhibited the NSCLC stem cell properties and radiation resistance. Collectively, CHIP promotes the ubiquitination of MAST1 by blocking the interaction of Hsp90*β* with MAST1, leading to decreased MAST1 protein stability, which suppressed NSCLC stem cell properties and radiation resistance.

## 1. Introduction

Non-small-cell lung cancer (NSCLC) is a major contributor to cancer mortality on a global scale, which is associated with a poor prognosis [1]. Radiotherapy is a significant therapeutic regimen in the treatment of NSCLC [2], but radiation resistance still poses a grave threat to survival of patients with NSCLC [3]. Of note, cancer stem cells (CSCs) are often found to be associated with radiation tolerance and recurrence in lung cancer [4]. CSCs are regarded as a small subgroup of heterogeneous cells in tumor tissues or cell lines with self-renewal and differentiation abilities, which are recognized to be accountable for tumorigenesis, recurrence, and therapeutic resistance [5, 6]. In this context, it is of significance to seek novel targets to orchestrate phenotypes of CSCs for the control of radiation resistance in NSCLC.

Previous evidence has uncovered the association of carboxyl terminal of Hsp70-interacting protein (CHIP) expression with survival time of patients with NSCLC, suggesting it as an important independent prognostic biomarker [7]. CHIP is an E3 ubiquitin ligase playing an important role in the protein quality control system through transfer of the folding-refolding machinery balance to the degradative pathway [8]. Interestingly, CHIP has been found to suppress CSC properties in breast cancer cells [9]. However, the effects of CHIP on NSCLC stem cells have not been identified. The GEO- and GeneCards database-based bioinformatics prediction of the current study suggested that CHIP was associated with radiation resistance of NSCLC stem cells.

Importantly, CHIP functions as a negative cochaperone for heat-shock protein 90 (Hsp90) [10]. Heat shock proteins (Hsps) are identified as molecular chaperones aiding in appropriate protein folding and refolding in cells against radiotherapy [11]. Hsp90 inhibitors have been highlighted to be of therapeutic use in NSCLC [12]. Intriguingly, highly expressed Hsp90*β* was shown to be a potential factor contributing to poor survival in NSCLC patients [13]. Notably, CHIP could negatively regulate microtubule-associated serine/threonine kinase 1 (MAST1) to induce MAST1 ubiquitination and degradation, and Hsp90*β* could interact with and stabilize MAST1 in cisplatin-resistant cancer cells [14]. MAST1 was reported to induce cisplatin resistance in human cancers through rewiring MEK activation independent of cRaf [15]. Nevertheless, there is a paucity of study regarding the role of Hsp90*β* and MAST1 in the NSCLC stem cell properties and radiation resistance. Considering the aforementioned findings, a hypothesis was proposed in this study that CHIP is likely to regulate radiation resistance in NSCLC stem cells through mediation of the Hsp90*β* and MAST1.

## 2. Materials and Methods

### 2.1. Ethical Approval

This study was approved by the Ethics Committee of Affiliated Cancer Hospital of Zhengzhou University, Henan Cancer Hospital. All the patients provided written informed consents. Animal experiments were approved by the Animal Ethics Committee of Affiliated Cancer Hospital of Zhengzhou University, Henan Cancer Hospital.

### 2.2. Bioinformatics Analysis

Through the “limma” package in R language, differential analyses were performed on the mRNA expression microarrays GSE84108 (including 3 Lewis lung cancer cell samples from mice treated with low dose radiotherapy and 2 samples from those treated with high dose radiotherapy) in the GEO database. Through the database GeneCards, “CSC”-related genes were retrieved and downloaded with relevance score ≥ 20 as the screening condition. The jvenn tool was used to intersect the differential genes in the GSE84108 microarray with the results screened from the GeneCards database to identify the candidate genes. Bioinformatics tools were used for gene functional enrichment analysis of the candidate genes. The GEPIA database was used for the analysis of the correlation of the key genes with the tumor stem cell markers in lung cancer.

### 2.3. Clinical Tissue Sample Collection

Totally, 36 NSCLC patients treated surgically in Affiliated Cancer Hospital of Zhengzhou University, Henan Cancer Hospital, were selected, and the NSCLC tissues and matched adjacent normal tissues were obtained during surgery. Tissues were fixed in formalin and paraffin-imbedded for histopathological diagnosis. All tissue sections were evaluated by experienced pathologists and diagnosed according to the diagnostic criteria of NSCLC issued by the World Health Organization. CHIP expression in cancer tissues of NSCLC patients was measured by reverse transcription-quantitative polymerase chain reaction (RT-qPCR), and the CHIP expression was classified as high and low expression based the median value of expression.

### 2.4. Immunohistochemical Staining

Cancer tissue blocks were fixed in 4% paraformaldehyde for 12 h. The tissues were boiled in 0.01 M citrate buffer for 15-20 min and cooled to room temperature. Goat serum sealing solution was added to the tissues, which were allowed to rest for 20 min at room temperature. Primary antibodies to Hsp90*β* (ab32568, 1 : 200, Abcam, Cambridge, MA) and MAST1 (NBP1-81453, 1 : 200, Novus Biologicals, Littleton, CO) were used to incubate the tissues for 1 h. Next, 30 *μ*L secondary antibody goat anti-rabbit against immunoglobulin G (IgG) (ab6721, 1 : 2000, Abcam) was added to incubate the tissues at room temperature for 1 h. The tissues were dropped with streptavidin-peroxidase and allowed to rest at 37°C for 30 min. After DAB coloration for 5 min, hematoxylin counterstaining was performed for 2 min. Images were captured and observed under a microscope.

### 2.5. Cell Culture and Lentiviral Transduction

Human embryonic kidney cell HEK293T (CL-0005) and human NSCLC cell A549 (CL-0016) were purchased from Procell (Wuhan, China). A549 cells were cultured using Dulbecco's modified Eagle medium (DMEM)/F12 (Gibco) containing 10% fetal bovine serum (FBS, Gibco) and HEK293T using DMEM (Gibco) containing 10% FBS (Gibco) at 37°C with 5% CO_2_ in an incubator with 95% saturation humidity. Cells were subcultured at a growth density of about 90%.

For the construction of lentiviruses, the overexpression vector pHAGE-CMV-MCS-IRES-ZsGreen and helper plasmids pSPAX2 and pMD2.G were cotransduced into 293 T cells. The supernatants were collected 48 h after cell culture and filtered after centrifugation with a 0.45 *μ*m filter to collect virus. Centrifugation and concentration were performed again after 72 h for viral collection; the two groups of viruses were mixed, and titers were determined.

CD133^+^ cells derived from NSCLC patient cancer tissues and A549 cell line were subjected to lentiviral transduction: vector (negative control (NC) lentiviral vector), oe-CHIP (lentiviral vector overexpressing CHIP), oe-CHIP+oe-Hsp90*β* (lentiviral vector overexpressing CHIP and Hsp90*β*), and oe-CHIP+oe-MAST1 (lentiviral vector overexpressing CHIP and MAST1).

When the cells grew in the logarithm phase, they were detached with trypsin, triturated into 5 × 10^4^/mL cell suspension, and seeded in 6-well plates at 2 mL per well, followed by incubation at 37°C overnight. At 48 h after transduction, GFP expression efficiency was observed under the fluorescence microscopy. Stably transduced cell line was constructed; 72 h after viral infection, complete medium containing 2 *μ*g/mL puromycin was used for further culture for 5 days. The expression of relevant genes in each cell group was determined by RT-qPCR.

### 2.6. Flow Cytometric Sorting of CD133^+^ Cells

Cancer tissues from NSCLC patients were prepared into single-cell suspension using tumor dissociation kits (130-095-929, Miltenyi Biotech GmbH, Bergisch-Gladbach, Germany), and CD133^+^ cells were then isolated from the single-cell suspension using CD133 cell isolation kits (130-100-857, Miltenyi Biotech GmbH).

A549 cells were typically detached into single-cell suspension and incubated with magnetic beads bound to CD133 antibody (130-097-049, Miltenyi Biotech GmbH). With the use of the QuadroMACS™ Separator and Starting Kits (130-1402-109, Miltenyi Biotech GmbH), CD133^+^ cells were isolated, which were then cultured in DMEM/F12 supplemented with 20 ng/mL epidermal growth factor (EGF) (PHG0311, Gibco) and 20 ng/mL basic fibroblast growth factor (bFGF) (13256-029, Gibco). The purity of the samples was assessed by flow cytometry. The percentage of CD133^+^ cells reaching more than 90% demonstrated successful sorting.

### 2.7. Sphere Formation Assay

A total of 10^3^ CD133^+^ cells were isolated from NSCLC tissues and the lung cancer cell line A549 and cultured at 37°C with 5% CO_2_ in serum-free DMEM/F12 containing 100 U/mL penicillin and 100 *μ*g/mL streptomycin, 20 ng/mL EGF (PHG0311, Gibco), 10 ng/mL bFGF (13256-029, Gibco), 5 *μ*g/mL insulin (HY-P1156, MedChemExpress, Monmouth Junction, NJ), 0.4% bovine serum albumin (BSA) (A1933, Sigma-Aldrich Chemical Company, St. Louis, MO), and 2% B27 (17504044, Gibco). After 10 days of culturing, the sphere number was counted under an inverted microscope (Olympus, Tokyo, Japan, IX73).

### 2.8. RT-qPCR

Total tissue or cell RNA was strictly extracted using TRIZOL (15596018, Invitrogen, Carlsbad, CA) according to the instructions, and the RNA concentration was determined. Primers used for this study were synthesized by Takara (Dalian, China) and designed in NCBI (Supplementary Table 1). Reverse transcription was performed according to the instructions of complementary DNA (cDNA) reverse transcription kits (K1622, Yaada, Beijing, China). A quantitative PCR instrument (ViiA 7, Life Technologies, Carlsbad, CA) was utilized for detection. With 2 *μ*g total cDNA used as the template and GAPDH as the reference primer, the 2^-△△Ct^ method was used to calculate the relative transcript level of the target genes [16].

### 2.9. Western Blot Assay

Total protein was extracted by the radioimmunoprecipitation assay (RIPA) lysis buffer (P0013C, Beyotime, Shanghai, China), and the protein concentration was determined using the BCA kits (20201ES76, Yeasen Company, Shanghai, China). The proteins were separated by polyacrylamide gel electrophoresis and transferred onto a polyvinylidene fluoride membrane (IPVH85R, Millipore, Billerica, MA) via wet transfer. The protein was then blocked with 5% BSA for 1 h at room temperature, followed by overnight incubation at 4°C with rabbit anti-CHIP (ab134064, 1 : 1000, Abcam), rabbit anti-Hsp90*β* (ab32568, 1 : 1000, Abcam), mouse anti-MAST1 (sc-373845, 1 : 1000, Santa Cruz Biotechnology, Santa Cruz, CA), or rat anti-ubiquitin (3936S, 1 : 1000, Cell Signaling Technologies, Beverly, MA). Horseradish peroxidase- (HRP-) labeled goat anti-rabbit against IgG (ab6721, Abcam, UK) or goat anti-rat against IgG (ab6789, Abcam) was added to incubate the samples for 1 h at room temperature. Following ECL development, the ImageJ software (National Institutes of Health, Bethesda, MA) was used for protein quantification analysis as normalized to the internal reference glyceraldehyde-3-phosphate dehydrogenase (GAPDH).

For the cycloheximide (CHX) tracking analysis, the transduced CD133^+^ cells were treated with 25 *μ*g/mL CHX (5087390001, Sigma-Aldrich, a protein synthesis inhibitor) for 12 h. The final concentration of CHX was 25 *μ*g/mL, and cells were cultured for 12 h under normal culture conditions, followed by Western blot assay [17].

### 2.10. Immunofluorescence

Cells were fixed with 4% paraformaldehyde and permeabilized with 0.2% Triton X-100 for 5 min. The sections were blocked with 10% normal goat serum. Rabbit anti-*γ*-H2AX antibody (9718, Cell Signaling Technologies, 1: 100) was added to incubate the samples overnight at 4°C. The next day, the secondary antibody Alexa Fluor 555-conjugated goat anti-rabbit against IgG (ab150078, 1: 300, Abcam) was added to incubate the samples at room temperature for 1 h. Following DAPI (C1006, Beyotime) staining, the sections were sealed and photographed under a microscope (BX63, Olympus).

### 2.11. Coimmunoprecipitation (Co-IP)

Cells were lysed in IP lysis buffer, and cell debris were removed by centrifugation. Cell lysates were incubated overnight with rat anti-MAST1 (sc-373845, 1 : 100, Santa Cruz Biotechnology) at 4°C. With IgG (ab281590, Abcam) as a NC, Protein A/G beads (Santa Cruz Biotechnology) were added to incubate the samples at 4°C for 2 h. The beads were boiled at 100°C for 5 min. The ubiquitination of MAST1 and expression of Hsp90*β*, MAST1, and CHIP were determined by Western blot assay [14].

### 2.12. Cell Survival Assessment after Radiation

A total of 1000 cells were seeded in a 6 cm dish and were exposed to a range of gamma rays (Shepherd Mark 1 68 Irradiator, Cs-137 source with a dose rate of 3.5 Gy/min, 2-8 Gy) 24 h after transduction. The medium was renewed periodically, and cells were cultured for 14 days until colonies were obviously large enough. Positive colonies were defined as those with more than 50 cells. Cells were fixed with 4% paraformaldehyde (P0099, Beyotime) for 10 min and then stained with 0.1% crystal violet (C0121) for 30 min. Colonies were counted under an optical microscope.

### 2.13. Flow Cytometric Detection of Cell Apoptosis

Apoptosis was evaluated by Annexin V-FITC/PI double staining. Cells were seeded in 6-well plates at 2 × 10^5^ cells/well. Cells were irradiated (Shepherd Mark 1 68 Irradiator, Cs-137 source; dose rate of 3.5 Gy/min, 4 Gy) after 24 h of transfection, and the culture was continued for 72 h. Following trypsinization, cells were centrifuged at 800 g and the supernatant was discarded. The precipitates were washed twice with PBS, and the cells were resuspended in 500 *μ*L of binding buffer in accordance with the instructions of apoptosis detection kits (556547, BD Bioscience, Franklin Lakes, NJ). Furthermore, 5 *μ*L fluorescein isothiocyanate (FITC) and 5 *μ*L propidium iodide (PI) were added to the cells in the darkness, followed by incubation for 15 min. Apoptosis was determined by a flow cytometer (FACS Calibur, BD Bioscience).

### 2.14. Tumor Xenograft Experiments in Nude Mice

Healthy nude mice (aged 6-8 weeks old) were raised in a specific-pathogen-free animal laboratory (humidity = 60% ~ 65%, temperature = 22 ~ 25°C), with free access to food and water under 12 h light/dark cycles. The mice were acclimatized for one week before experiments.

Mice were randomly assigned into different groups with 5 mice in each group. A total of 1 × 10^6^ stably transduced CD133^+^ cells derived from the A549 cells were injected subcutaneously into nude mice. The general situations and the tumor inoculation sites of nude mice were observed every day. When the tumor volume reached approximately 100 mm^3^, gamma rays (Shepherd Mark 1 68 Irradiator, Cs-137 source, dose rate of 3.5 Gy/min) were locally given to the mice at a total dose of 20 Gy every five days at 10 Gy each time. Tumor volumes were then measured and calculated every 2 days. Tumor volume was calculated as *V* = (length and width^2^)/2.

### 2.15. Statistical Analysis

All data were processed using SPSS 21.0 statistical software (SPSS, IBM, Armonk, NY). Measurement data obtained from three independent experiments were expressed in the form of mean ± standard deviation. Comparisons between two groups were conducted by an unpaired *t-*test, and comparisons between multiple groups were performed by one-way analysis of variance (ANOVA). *p* < 0.05 indicated significant differences.

## 3. Results

### 3.1. E3 Ubiquitin Ligase CHIP Was Poorly Expressed in NSCLC Stem Cells

**Figure 1:**
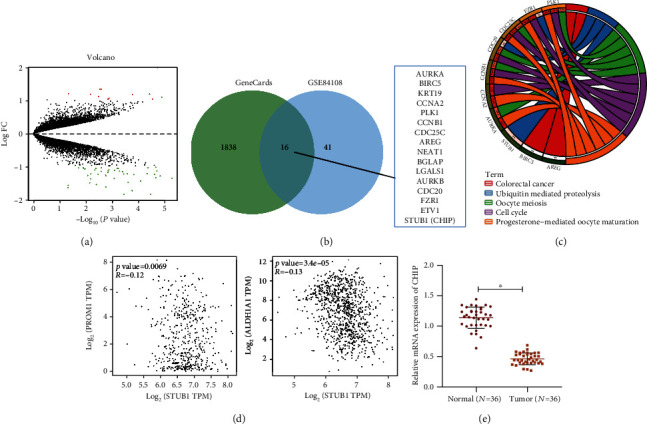
Expression of the E3 ubiquitin ligase CHIP in NSCLC. (a) The volcano map of lung cancer radiotherapy-related microarray GSE84108 in the GEO database. Red indicates the upregulated genes, and green indicates the downregulated genes. (b) The Venn plot of the screening results from GeneCards database and the results of the microarray GSE84108 analysis. (c) The results showing KEGG functional enrichment analysis of candidate genes in the GeneCards database analysis and the microarray GSE84108 analysis. (d) The correlation of STUB1 (CHIP) expression with the CSC markers CD133 (PROM1) and ALDH1A1 (ALDH1) in lung cancer analyzed by the GEPIA database. (e) CHIP expression in cancer and adjacent normal tissues of NSCLC patients (*n* = 36) determined by RT-qPCR. ^∗^*p* < 0.05.

To explore the specific mechanism of radiation resistance in NSCLC stem cells, we retrieved lung cancer-related microarray GSE84108 through the GEO database. Based on the comparison of the transcriptomes of NSCLC cell lines treated with high and low dose of radiotherapy, 57 radiotherapy-related genes were obtained (Figure 1(a)). A total of 1854 tumor stem cell-related genes (relevance score = 20) were retrieved from the GeneCards database. After the two datasets were intersected, 16 candidate genes were obtained (Figure 1(b)). Functional enrichment analysis of candidate genes by online tools found that the candidate genes were mainly enriched in the signaling pathways such as progesterone-mediated oocyte maturation (hsa04914), cell cycle (hsa04110), oocyte meiosis (hsa04114), ubiquitin mediated proteolysis (hsa04120), and colorectal cancer (hsa05210) (Figure 1(c)).

Multiple studies have shown that ubiquitination-mediated protein degradation plays vital roles in tumor stem cell self-renewal and radiation resistance [18–20]. It appears from Figure 1(c) that the genes enriched in the ubiquitin mediated proteolysis signaling pathway included the E3 ubiquitin ligase STUB1 (CHIP). According to previous evidence, CHIP inhibits the properties of CSCs [9, 21] and the development of lung cancer [22]. We found by GEPIA data analysis that CHIP expression was negatively associated with the expression of the CSC markers CD133 (PROM1) and ALDH1A1 (ALDH1) in NSCLC (Figure 1(d)). Therefore, we selected CHIP as the target gene for this study.

Next, we performed RT-qPCR on NSCLC and adjacent normal tissues. The results displayed that CHIP was significantly downregulated in cancer tissues of NSCLC patients as compared with that in adjacent normal tissues (Figure 1(e)). The above results confirmed that CHIP was poorly expressed in NSCLC tissues.

**Figure 2:**
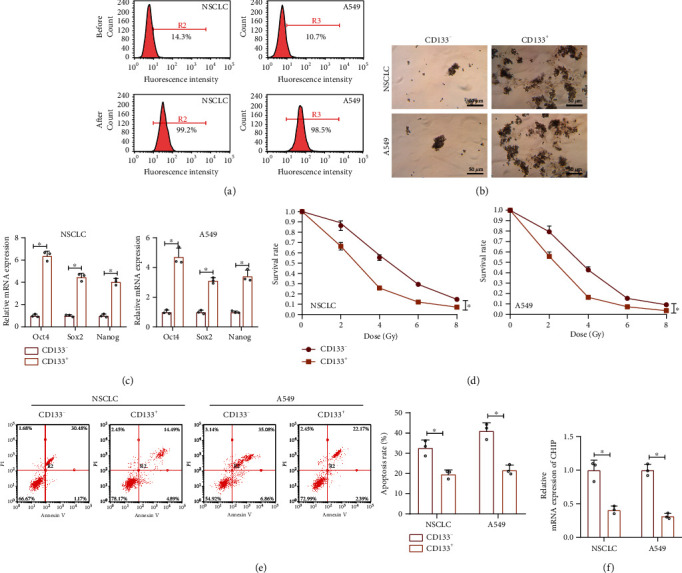
Expression of the E3 ubiquitin ligase CHIP in NSCLC stem cells. (a) Flow cytometric sorting of CD133^+^ cells from the cancer tissues of NSCLC patients (*n* = 7) and the lung cancer cell line A549. The isolated CD133^+^ cells were stained with anti-CD133^+^ antibody. (b) Representative images of sphere formation after 7 days of sphere culture of CD133^−^ and CD133^+^ NSCLC and A549 cells. (c) The expression of stemness-related transcription factors (Oct4, SOX2, and Nanog) in CD133^−^ and CD133^+^ NSCLC and A549 cells detected by RT-qPCR. (d) Clonal survival rate quantitation of radiation-exposed CD133^−^ and CD133^+^ NSCLC and A549 cells. (e) Apoptosis of CD133^−^ and CD133^+^ NSCLC and A549 cells detected by Annexin V/PI double staining. (f) The CD133 expression in CD133^−^ and CD133^+^ NSCLC and A549 cells detected by RT-qPCR. ^∗^*p* < 0.05*vs.* CD133^−^ cells. Cell experiments were independently repeated three times.

Then, we explored the relationship between CHIP and radiation resistance in NSCLC stem cells. First, we isolated CD133^+^ cells from the NSCLC tissues and the A549 cell line. Flow cytometry results showed that the isolated CD133^+^ cells had a purity higher than 90% (Figure 2(a)). The sphere formation assay showed that CD133^+^ cells could form floating spheroids (Figure 2(b)). Relative to the CD133^−^ cells, CD133^+^ cells had significantly increased expression of stemness-related transcription factors (Oct4, SOX2, and Nanog) (Figure 2(c)). These results suggested that CD133^+^ cells present properties of CSCs.

Furthermore, we also demonstrated that, relative to CD133^−^ cells, CD133^+^ under the same radiation conditions showed higher cell survival and lower apoptotic rates, suggesting that CD133^+^ cells were more resistant to radiation compared with CD133^−^ cells (Figures 2(d) and 2(e)). The RT-qPCR results also revealed that CHIP was downregulated in CD133^+^ cells (Figure 2(f)).

Collectively, E3 ubiquitin ligase CHIP is downregulated in NSCLC stem cells and may be associated with radiation resistance in NSCLC.

### 3.2. Overexpression of CHIP Inhibited the NSCLC Stem Cell Properties and Radiation Resistance

**Figure 3:**
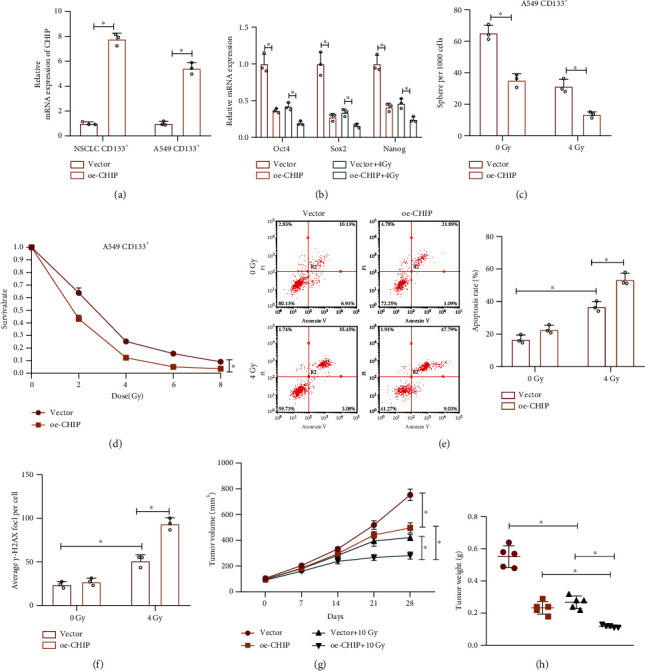
Overexpression of CHIP inhibits the NSCLC stem cell properties and radiation resistance. (a) RT-qPCR analysis for CHIP expression in the CD133^+^ NSCLC and A549 cells treated with lentiviral oe-CHIP. (b) RT-qPCR analysis for the expression of stemness-related transcription factors (Oct4, SOX2, and Nanog) in CD133^+^ A549 cells in response to CHIP overexpression alone or combined with 4 Gy radiation. (c) Sphere formation analysis after 7 days of sphere culture in CD133^+^ A549 cells in response to CHIP overexpression alone or combined with 4 Gy radiation. (d) Cell survival analyzed by colony formation assay in CD133^+^ A549 cells exposed to different doses of radiation after 9 days of culture. (e) Annexin V/PI double staining for detection of apoptosis of CD133^+^ A549 cells in response to CHIP overexpression alone or combined with 4 Gy radiation. (f) Immunofluorescence detection of the number of *γ*-H2AX foci in CD133^+^ A549 cells in response to CHIP overexpression alone or combined with 4 Gy radiation. (g) The quantitation of tumor volume in nude mice subcutaneously injected with CD133^+^ A549 cells in response to CHIP overexpression alone or combined with 4 Gy radiation (*n* = 5). (h) Statistics of tumor weight of nude mice in response to CHIP overexpression alone or combined with 4 Gy radiation (*n* = 5). ^∗^*p* < 0.05. Cell experiments were independently repeated three times.

We than moved to investigate the effect of CHIP on NSCLC stem cells. Lentiviral oe-CHIP was introduced in CD133^+^ cells, and the efficiency was verified by RT-qPCR (Figure 3(a)). As shown in Figures 3(b) and 3(c) and Supplementary Figure 1A, B, overexpression of CHIP inhibited the expression of stemness-related transcription factors (Oct4, SOX2, and Nanog) and sphere formation capacity in CD133^+^ cells, suggesting that CHIP could inhibit the self-renewal ability of NSCLC stem cells.

Meanwhile, we found that a further decrease was noted in stemness-related transcription factor expression and sphere formation capacity when CHIP-overexpressing cells were irradiated (Figures 3(b) and 3(c), Supplementary Figure 1A, B). Moreover, overexpression of CHIP inhibited cell survival and promoted cell apoptosis under radiation (Figures 3(d) and 3(e), Supplementary Figure 1C, D). Moreover, overexpression of CHIP promoted the increase in the number of *γ*-H2AX foci caused by radiation (Figure 3(f), Supplementary Figure 1E), indicating that upregulation of CHIP promoted the radiation-induced cellular DNA damage. Therefore, restored CHIP inhibited radiation resistance in NSCLC stem cells.

Furthermore, we constructed lung cancer tumor xenograft models in nude mice and further irradiated them to observe tumor formation. According to results in Figures 3(g) and 3(h), upregulated CHIP or radiation inhibited tumor growth, and overexpression of CHIP could promote the inhibitory effect of radiation on tumor growth.

Collectively, overexpressed CHIP can inhibit *in vivo* tumorigenic ability and radiation resistance of NSCLC stem cells.

### 3.3. CHIP Ubiquitylated MAST1 and Suppressed Its Protein Stability

**Figure 4:**
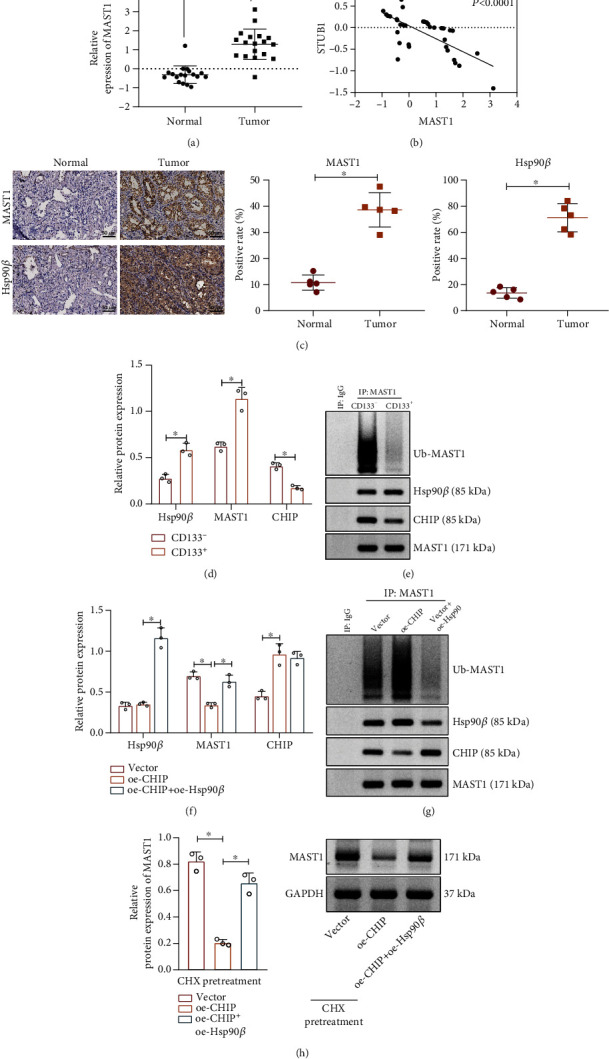
CHIP ubiquitylates MAST1 and suppresses its protein stability. (a) MAST1 expression in the NSCLC-related microarray data GSE74706 (*n* = 18). (b) The correlation between MAST1 and CHIP expression in lung cancer patients of GSE74706 microarray (*n* = 18). (c) The expression of Hsp90*β* and MAST1 in cancer and adjacent normal tissues of NSCLC patients determined by immunohistochemical staining (*n* = 5). (d) Western blot assay to determine the expression of Hsp90*β*, MAST1, and CHIP in CD133^−^ and CD133^+^ A549 cells. (e) The interaction of CHIP, Hsp90*β*, and MAST1 and the ubiquitination of MAST1 in CD133^−^ and CD133^+^ A549 cells detected by Co-IP assay. (f) Western blot assay for Hsp90*β*, MAST1, and CHIP in CD133^+^ A549 cells in response to overexpression of CHIP and Hsp90*β* alone or in combination. (g) Co-IP assay for interaction of CHIP, Hsp90*β*, and MAST1 and the ubiquitination of MAST1 in CD133^+^ A549 cells. (h) Western blot assay for MAST1 expression in CD133^+^ A549 cells after treatment with CHX (concentration of 25 *μ*g/mL, an inhibitor of protein synthesis) for 12 h. ^∗^*p* < 0.05. Cell experiments were independently repeated three times.

Evidence exists reporting that the interaction of Hsp90*β* and MAST1 blocked MAST1 degradation by CHIP ubiquitination [14]. From a differential analysis of the lung cancer-related microarray GSE74706, we found that MAST1 was highly expressed in lung cancer samples as compared with the normal samples (Figure 4(a)). Besides, MAST1 was significantly negatively associated with CHIP expression in lung cancer (Figure 4(b)). Previous evidence has indicated that MAST1 may act as an activator of the MEK1 and MAPK cascade that in turn drives cisplatin resistance in human cancers [15]. Thus, we hypothesized that the E3 ubiquitin ligase CHIP may regulate the NSCLC stem cell properties and radiation resistance by regulating Hsp90*β*-MAST1 signaling.

Next, immunohistochemistry revealed that Hsp90*β* and MAST1 were highly expressed in cancer tissues of NSCLC patients (Figure 4(c)). Meanwhile, we also found that, relative to those in CD133^−^ cells, Hsp90*β* and MAST1 were highly expressed in CD133^+^ cells, accompanied with enhanced interaction, decreased ubiquitination of MAST1, and decreased interaction between MAST1 and CHIP (Figures 4(d) and 4(e)).

Next, CD133^−^ cells derived from A549 cells were transduced with the corresponding plasmids. As illustrated in Figures 4(f) and 4(g), treatment of oe-CHIP or oe-CHIP+oe-Hsp90*β* resulted in increased CHIP expression. The treatment of oe-CHIP diminished interaction between MAST1 protein and Hsp90*β*, accompanied by increased ubiquitination and interaction with CHIP. Relative to oe-CHIP alone, oe-CHIP+oe-Hsp90*β* elevated protein levels of Hsp90*β* and MAST1, enhanced interaction of MAST1 with Hsp90*β*, and diminished ubiquitination of MAST1 and the interaction of MAST1 with CHIP. In addition, CHX was used to inhibit protein synthesis, and the Western blot assay results displayed that upregulation of CHIP promoted MAST1 protein degradation, while Hsp90*β* overexpression counterweighed its effect (Figure 4(h)).

Thus, the E3 ubiquitin ligase CHIP promoted MAST1 ubiquitination by blocking Hsp90*β* interaction with MAST1, thereby inhibiting MAST1 protein stability.

### 3.4. CHIP Suppressed NSCLC Stem Cell Properties and Radiation Resistance through the Inhibition of MAST1 Protein Stability

**Figure 5:**
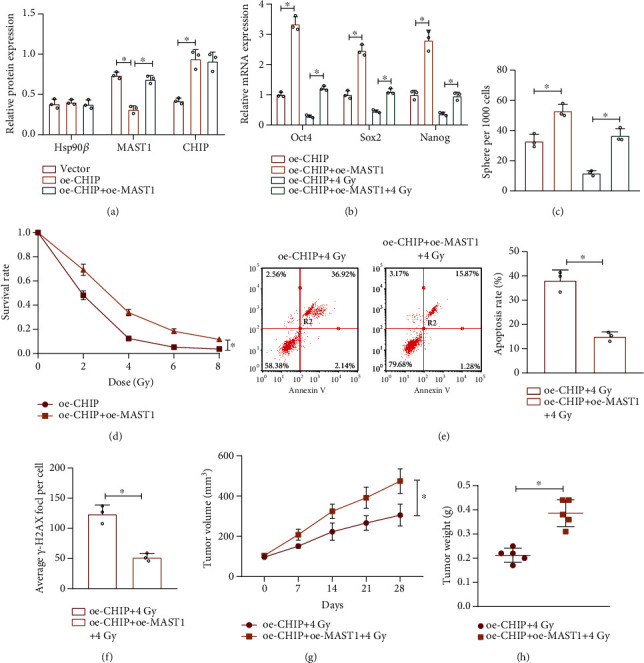
CHIP inhibits the NSCLC stem cell properties and radiation resistance through the inhibition of MAST1 protein stability. (a) Western blot assay to determine the expression of CHIP, MAST1, and Hsp90*β* in CD133^+^ A549 cells in response to overexpression of CHIP and MAST1 alone or in combination. (b) RT-qPCR analysis for expression of stemness-related transcription factors (Oct4, SOX2, and Nanog) in irradiated/unirradiated (4 Gy) CD133^+^ A549 cells in response to overexpression of CHIP and MAST1 alone or in combination. (c) Sphere formation analysis after 7 days of sphere culture of irradiated/unirradiated (4 Gy) CD133^+^ A549 cells in response to overexpression of CHIP and MAST1 alone or in combination. (d) Cell survival analysis of CD133^+^ A549 cells exposed to different doses of radiation by colony formation assay after 9 days of culture. (e) Annexin V/PI double staining for detection of apoptosis of irradiated (4 Gy) CD133^+^ A549 cells in response to overexpression of CHIP and MAST1 alone or in combination. (f) Immunofluorescence detection of the number of *γ*-H2AX foci in irradiated (4 Gy) CD133^+^ A549 cells in response to overexpression of CHIP and MAST1 alone or in combination. (g) The quantitation of tumor volume in nude mice subcutaneously injected with CD133^+^ A549 cells in response to overexpression of CHIP and MAST1 alone or in combination as well as irradiation (4 Gy) (*n* = 5). (h) Statistics of tumor weight of nude mice in response to overexpression of CHIP and MAST1 alone or in combination as well as irradiation (4 Gy) (*n* = 5). ^∗^*p* < 0.05. Cell experiments were independently repeated three times.

Furthermore, we overexpressed CHIP or MAST1 in CD133^+^ cells derived from A549 cell line and NSCLC samples, respectively, in order to explore the effect of CHIP-mediated MAST1 protein stability in NSCLC stem cells. Increased CHIP expression was shown by Western blot assay in response to oe-CHIP or oe-CHIP+oe-MAST1. MAST1 expression was reduced in response to oe-CHIP, while additional treatment of oe-MAST1 increased MAST1 relative to oe-CHIP alone (Figure 5(a), Supplementary Figure 2A). As shown in Figures 5(b) and 5(c) and Supplementary Figure 2B, C, overexpression of MAST1 reversed the CHIP overexpression-induced inhibition in the expression of stemness-related transcription factors (Oct4, SOX2, and Nanog) and suppression of sphere formation capacity in CD133^+^ cells. This result suggested that CHIP inhibited the self-renewal ability of NSCLC stem cells by downregulating MAST1 protein stability.

Meanwhile, we also found that additional overexpression of MAST1 impaired the CHIP overexpression-induced inhibition in stemness-related transcription factor expression and sphere formation capacity (Figures 5(b) and 5(c), Supplementary Figure 2B, C). In addition, cell survival was increased in response to oe-CHIP+oe-MAST1 as compared with that in response to CHIP overexpression alone (Figures 5(d) and 5(e), Supplementary Figure 2D, E). Moreover, overexpression of MAST1 notably reversed the promoting effect of CHIP overexpression on the number of radiotherapy-caused *γ*-H2AX foci (Figures 5(f), Supplementary Figure 2F). Overall, CHIP suppressed the radiation resistance in NSCLC stem cells by repressing MAST1 protein stability.

Furthermore, we constructed and irradiated lung cancer tumor xenograft models in nude mice, observed, and recorded tumor formation. As shown in Figures 5(g) and 5(h), cooverexpression of CHIP and MAST1 negated the radiation-induced inhibition on tumor growth relative to CHIP overexpression alone.

These results suggest that the overexpression of CHIP inhibits the *in vivo* tumorigenic ability and radiation resistance in NSCLC stem cells through suppression of MAST1 protein stability.

## 4. Discussion

**Figure 6:**
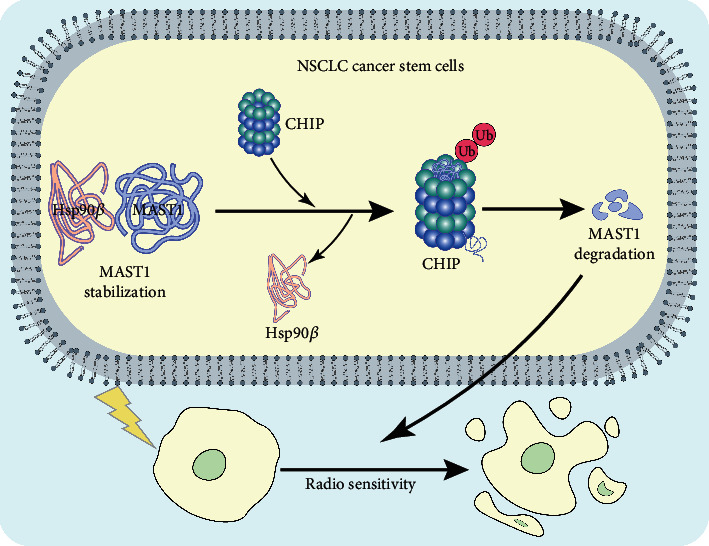
Plot of the molecular mechanism of E3 ubiquitin ligase CHIP in radioresistance of NSCLC stem cells. E3 ubiquitin ligase CHIP promotes the ubiquitination of MAST1 by blocking the interaction of Hsp90*β* with MAST1, leading to a decrease in MAST1 protein stability, thus inhibiting NSCLC stem cell properties and promoting radiosensitivity.

Drugs or radiotherapy could exert their effects on cells by changing the expression or protein levels of some genes. For example, demethylzeylasteral could increase the expression of apoptotic genes and reduce the expression of antiapoptotic genes in liver cancer stem cells [23]. In addition, radiation treatment can promote the radiation resistance of colon cancer stem cells through the activation of JAK2/STAT3/CCND2 signaling pathway [24]. It has also been reported that radiotherapy could change the expression of Caveolin-1, which could further affect the radiation response of pancreatic cancer and lung cancer [25]. Proteomics has indicated that a large number of proteins have been altered after radiotherapy treatment [26]. Therefore, based on differential analysis in NSCLC cell lines exposed to low-dose and high-dose radiation, radiotherapy-related genes were obtained for further verification experiments. We retrieved lung cancer-related microarray GSE84108 through the GEO database and obtained 57 genes potentially related to radiation resistance by comparing with the transcriptomes of NSCLC cell lines treated with high and low dose of radiotherapy. In the present study, we revealed that E3 ubiquitin ligase CHIP suppressed the properties and resistance of NSCLC stem cells by mediating the Hsp90*β*-MAST1 axis (Figure 6).

We validated the downregulation of CHIP in NSCLC stem cells and further found that overexpression of CHIP inhibited the NSCLC stem cell properties and radiation resistance. In consistency with our finding, a previous study has also shown the decreased CHIP expression in NSCLC tissues as well as its correlation with clinical stages and metastasis of this malignancy [7]. Moreover, a positive relationship between CHIP expression and the longer overall survival of NSCLC patients has been demonstrated by Kim et al. [27]. Besides, CHIP is reported to repress tumor metastasis in lung cancer by polyubiquitination of ovarian tumor domain-containing protein 3 to promote its degradation [28]. Interestingly, it was revealed that CHIP could alleviate the stemness of thyroid cancer cells through downregulation of Oct4 protein stability [29]. Additionally, CHIP mediates CD166 protein stability via the ubiquitin proteasome system, thereby repressing the properties of CSCs in head and neck cancer [21]. Overall, these data can support our finding in regard to the inhibitory role of overexpressed CHIP in NSCLC stem cells.

Mechanistically, our study further found that CHIP could block the interaction of Hsp90*β* with MAST1 to promote ubiquitination of MAST1, thereby inhibiting the protein stability of MAST1 in NSCLC stem cells. Besides, downregulation of MAST1 protein stability could suppress the NSCLC stem cell properties and radiation resistance. CHIP can bind to Hsp90 chaperones via the tetratricopeptide repeat domain and serves as an E3 ubiquitin ligase through a modified RING finger domain (U-box), and the combined two domains can allow CHIP to network chaperone compounds to the ubiquitin-proteasome system [10]. Hsp90 can collaborate with the cochaperone CHIP and target their bound substrate to degradation via ubiquitination [30]. Recent evidence suggests that upregulated expression of HSP90*β* indicates poor prognosis in NSCLC by regulating malignant biological behaviors of cancer cells [31, 32]. Interestingly, it has been reported that Hsp90 inhibitor NVP-AUY922 contributed to enhanced radiation sensitivity of lung cancer cells resistant to EGFR-tyrosine kinase inhibitors [33]. Moreover, disrupted Hsp90 function by panaxynol could aid in the reduction of sphere forming capacity of CSCs in NSCLC at nanomolar concentrations [34].

Moreover, the interaction of Hsp90*β* with MAST1 could repress MAST1 ubiquitination at lysines 317 and 545 by CHIP and curbed proteasomal degradation; combined inhibition of Hsp90 and MAST1 could further disrupt MAST1 activity, which enhances cisplatin-triggered tumor growth arrest [14]. Evidence has been presented demonstrating that MAST1 has the biological function of promoting the cancer stem cell properties and radiation resistance [15]. Besides, MAST1 has been suggested to function as an oncogenic driver to lung cancer [35]. MAST1 inhibitor lestaurtinib could diminish cisplatin-resistant tumor growth [36]. It has been suggested that activation of MEK1 plays a vital regulatory role in MAST1-mediated chemotherapy resistance of cancer cells [37]. We speculated that the activation of MEK1 may promote the NSCLC stem cell properties and radiation resistance induced by MAST1, which should be further explored.

## 5. Conclusion

Taken together, the present study demonstrated that E3 ubiquitin ligase CHIP blocks the interaction of Hsp90*β* with MAST1 to promote the ubiquitination of MAST1, which contributes to a decrease in MAST1 protein stability. Therefore, the properties and radiation resistance in NSCLC stem cells were repressed. This finding suggests a promising target for controlling the radiation resistance in NSCLC.

## Data Availability

The datasets used and/or analyzed during the current study are available from the corresponding author on reasonable request.

## Supplementary Materials

Supplementary 1 Supplementary Figure 1: the effect of overexpression of CHIP on the NSCLC stem cell properties and radiation resistance from NSCLC patients. (A) RT-qPCR analysis for expression of stemness-related transcription factors (Oct4, SOX2, and Nanog) in CD133^+^ cells from the cancer tissues of NSCLC patients (*n* = 7) in response to CHIP overexpression alone or combined with 4 Gy radiation. (B) Representative images of sphere formation and sphere formation rate statistics after 7 days of sphere culture of CD133^+^ cells from the cancer tissues in response to CHIP overexpression alone or combined with 4 Gy radiation. (C) Cell survival analysis by colony formation assay in CD133^+^ cells from the cancer tissues exposed to different doses of radiation after 9 days of culture. (D) Annexin V/PI double staining for detection of apoptosis of CD133^+^ cells from the cancer tissues in response to CHIP overexpression alone or combined with 4 Gy radiation. (E) Immunofluorescence detection of the number of *γ*-H2AX foci in CD133^+^ cells from the cancer tissues in response to CHIP overexpression alone or combined with 4 Gy radiation. ^∗^*p* < 0.05. Cell experiments were independently repeated three times.

Supplementary 2 Supplementary Figure 2: the CHIP/MAST1 axis affects the properties and radiation resistance of NSCLC stem cells derived from NSCLC patients. (A) Western blot assay for expression of stemness-related transcription factors (Oct4, SOX2, and Nanog) in CD133^+^ cells from the cancer tissues in response to overexpression of CHIP and MAST1 alone or in combination. (B) RT-qPCR analysis for expression of stemness-related transcription factors (Oct4, SOX2, and Nanog) in irradiated/unirradiated (4 Gy) CD133^+^ cells isolated from cancer tissues in response to overexpression of CHIP and MAST1 alone or in combination. (C) Representative images of sphere formation and sphere formation rate statistics after 7 days of sphere culture of irradiated/unirradiated (4 Gy) CD133^+^ cells from the cancer tissues in response to overexpression of CHIP and MAST1 alone or in combination. (D) Cell survival analysis by colony formation assay in CD133^+^ cells from the cancer tissues exposed to different doses of radiation after 9 days of culture. (E) Annexin V/PI double staining for detection of apoptosis of irradiated (4 Gy) CD133^+^ cells from the cancer tissues in response to overexpression of CHIP and MAST1 alone or in combination. (F) Immunofluorescence detection of the number of *γ*-H2AX foci in irradiated (4 Gy) CD133^+^ cells from the cancer tissues in response to overexpression of CHIP and MAST1 alone or in combination. ^∗^*p* < 0.05. Cell experiments were independently repeated three times.

Supplementary 3 Supplementary Table 1: primer sequences for RT-qPCR.
