# Supplementary figures and images for: E3 Ubiquitin Ligase CHIP Inhibits the Interaction between Hsp90β and MAST1 to Repress Radiation Resistance in Non-Small-Cell Lung Cancer Stem Cells
Source: Stem Cells Int. 2022 Sep 20;2022:2760899. doi: 10.1155/2022/2760899 (PMC9527118; doi:10.1155/2022/2760899)

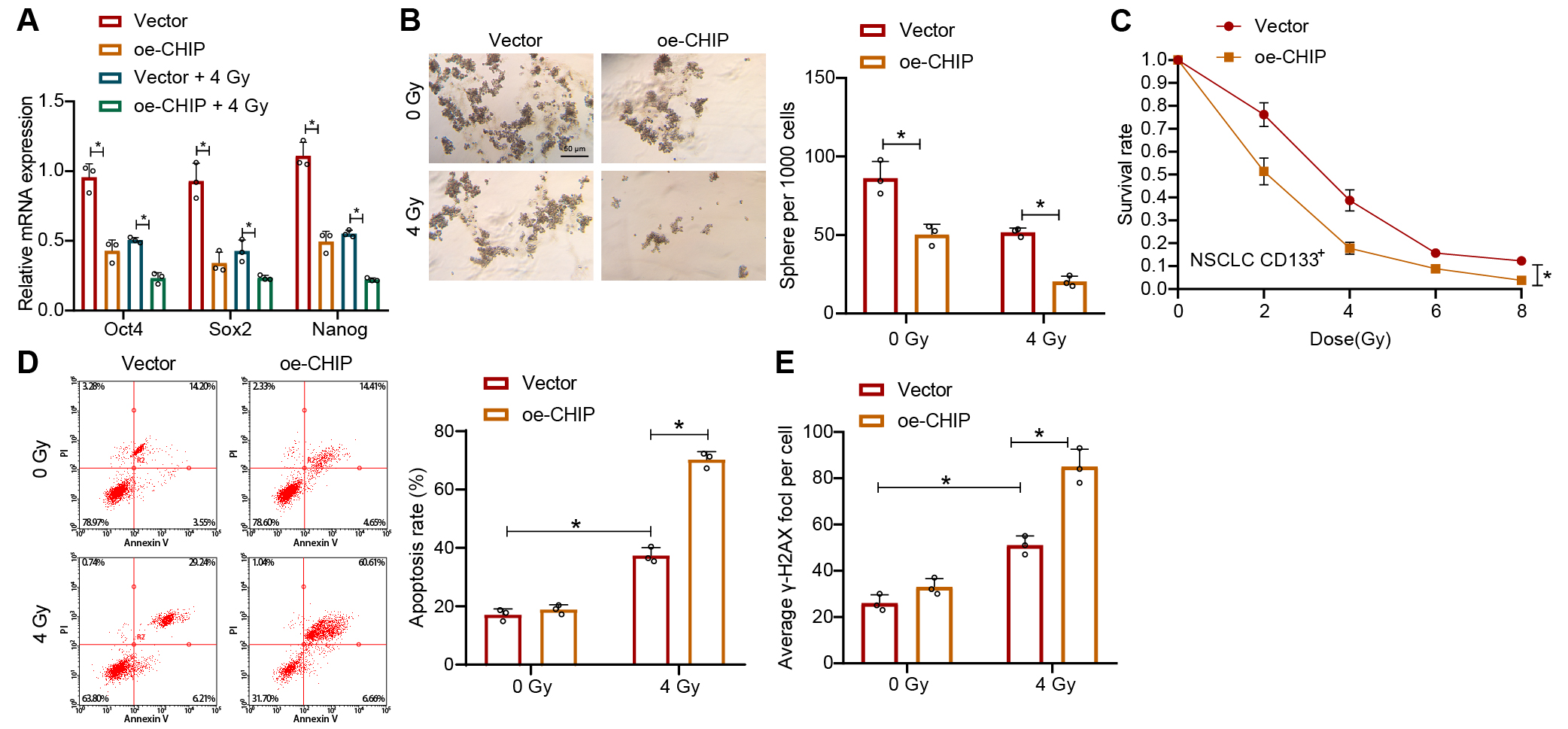

Supplement: Supplementary 1 — Supplementary Figure 1: the effect of overexpression of CHIP on the NSCLC stem cell properties and radiation resistance from NSCLC patients. (A) RT-qPCR analysis for expression of stemness-related transcription factors (Oct4, SOX2, and Nanog) in CD133+ cells from the cancer tissues of NSCLC patients (n = 7) in response to CHIP overexpression alone or combined with 4 Gy radiation. (B) Representative images of sphere formation and sphere formation rate statistics after 7 days of sphere culture of CD133+ cells from the cancer tissues in response to CHIP overexpression alone or combined with 4 Gy radiation. (C) Cell survival analysis by colony formation assay in CD133+ cells from the cancer tissues exposed to different doses of radiation after 9 days of culture. (D) Annexin V/PI double staining for detection of apoptosis of CD133+ cells from the cancer tissues in response to CHIP overexpression alone or combined with 4 Gy radiation. (E) Immunofluorescence detection of the number of γ-H2AX foci in CD133+ cells from the cancer tissues in response to CHIP overexpression alone or combined with 4 Gy radiation. ∗p < 0.05. Cell experiments were independently repeated three times. [file 2760899.f1.jpg]

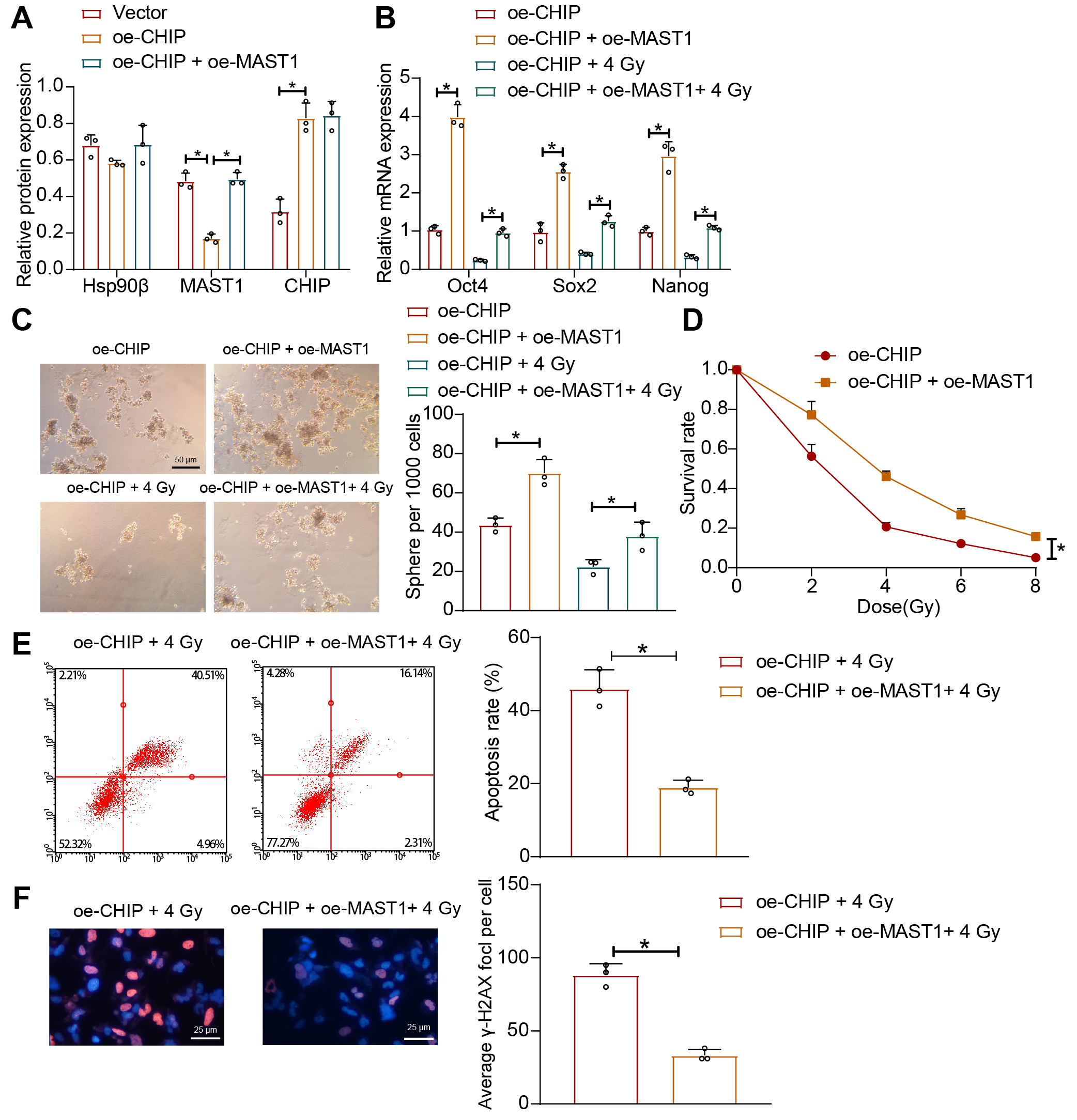

Supplement: Supplementary 2 — Supplementary Figure 2: the CHIP/MAST1 axis affects the properties and radiation resistance of NSCLC stem cells derived from NSCLC patients. (A) Western blot assay for expression of stemness-related transcription factors (Oct4, SOX2, and Nanog) in CD133+ cells from the cancer tissues in response to overexpression of CHIP and MAST1 alone or in combination. (B) RT-qPCR analysis for expression of stemness-related transcription factors (Oct4, SOX2, and Nanog) in irradiated/unirradiated (4 Gy) CD133+ cells isolated from cancer tissues in response to overexpression of CHIP and MAST1 alone or in combination. (C) Representative images of sphere formation and sphere formation rate statistics after 7 days of sphere culture of irradiated/unirradiated (4 Gy) CD133+ cells from the cancer tissues in response to overexpression of CHIP and MAST1 alone or in combination. (D) Cell survival analysis by colony formation assay in CD133+ cells from the cancer tissues exposed to different doses of radiation after 9 days of culture. (E) Annexin V/PI double staining for detection of apoptosis of irradiated (4 Gy) CD133+ cells from the cancer tissues in response to overexpression of CHIP and MAST1 alone or in combination. (F) Immunofluorescence detection of the number of γ-H2AX foci in irradiated (4 Gy) CD133+ cells from the cancer tissues in response to overexpression of CHIP and MAST1 alone or in combination. ∗p < 0.05. Cell experiments were independently repeated three times. [file 2760899.f2.jpg]
