# Supplementary material for: E3 Ubiquitin Ligase CHIP Inhibits the Interaction between Hsp90β and MAST1 to Repress Radiation Resistance in Non-Small-Cell Lung Cancer Stem Cells
Source: Stem Cells Int. 2022 Sep 20;2022:2760899. doi: 10.1155/2022/2760899 (PMC9527118; doi:10.1155/2022/2760899)
Supplement: Supplementary 3 — Supplementary Table 1: primer sequences for RT-qPCR. [file 2760899.f3.docx]

**Supplementary Table 1** Primer sequences for RT-qPCR

| Gene | Primer sequences (5'-3') |
| --- | --- |
| Oct4 | Forward: CTGGGTTGATCCTCGGACCT |
|  | Reverse: CCATCGGAGTTGCTCTCCA |
| SOX2 | Forward: GCCGAGTGGAAACTTTTGTCG |
|  | Reverse: GGCAGCGTGTACTTATCCTTCT |
| Nanog | Forward: TTTGTGGGCCTGAAGAAAACT |
|  | Reverse: AGGGCTGTCCTGAATAAGCAG |
| GAPDH | Forward: GGAGCGAGATCCCTCCAAAAT |
|  | Reverse: GGCTGTTGTCATACTTCTCATGG |
| CHIP | Forward: GGGCAATCGTCTGTTCGTG |
|  | Reverse: GGCGTAGGCTCAGCGTG |
